# Supplementary material for: Are carbon emissions trading and green financial instruments synergistic? -Comprehensive quantitative research based on content analysis
Source: PLoS One. 2024 Mar 7;19(3):e0298601. doi: 10.1371/journal.pone.0298601 (PMC10919601; doi:10.1371/journal.pone.0298601)
Supplement: S1 Data — (ZIP) [file pone.0298601.s001.zip › Data/地级市Z_F工作报告.zip!/╕╩╦α/┬ñ─╧╩╨/└┤╫╘╣1⁄2╓┌║┼í╛┬φ┐╦╩2╛▌═°í┐_2014.docx]

各位代表：

现在，我代表市人民政府向大会作工作报告，请予审议，并请各位政协委员和列席人员提出意见。

2013年工作回顾

2013年，面对宏观经济下行压力加大和自然灾害多发的严峻考验，我们在市委的坚强领导下，全面贯彻党的十八大及十八届二中、三中全会精神，认真落实省委、省政府重大决策部署和市委三届六次全委扩大会、市三届三次人代会精神，坚持以科学发展观为统领，牢牢把握稳中求进、好中求快的总基调，较好地完成了全年目标任务。预计全市完成生产总值252亿元，同比增长11%；完成固定资产投资537亿元，增长31%；完成大口径财政收入44.1亿元、公共财政预算收入21.48亿元，分别增长30.1%和34.1%；完成社会消费品零售总额65.4亿元，增长14%；城镇居民人均可支配收入16188元，增长15%；农民人均纯收入3613元，增长17%；新增城镇就业3.2万人，城镇登记失业率2.25%；居民消费价格总水平涨幅2.6%；单位生产总值能耗和主要污染物排放完成省上下达的控制指标。

一、经济总量和效益持续增长，产业发展步伐加快

坚持做大经济总量和提升发展质量并重，强化基础支撑与培育新的增长点并举，突出重点，破解难题，经济保持了持续、健康、快速的发展。农业上，继续实施科技增粮措施，粮食总产达到106万吨。核桃、花椒、油橄榄、中药材、苗木等特色产业新增面积41万亩，首次突破1000万亩大关，达到1015万亩。规模养殖比重达到34%，比上年增加3个百分点。完成经济林综合管理555万亩，嫁接换优420万株。新认定省级农业龙头企业4家，国家级1家，实现国家级龙头企业零的突破。新发展农民专合组织1508个。农村集体土地确权登记工作顺利推进，完成土地流转32.1万亩，林地流转31.1万亩。农村集贸市场和物流园区建设取得新进展。成功举办了全国退耕还林阶段性验收工作总结会，武都花椒、成县核桃、徽县苗木、宕昌中药材产销对接洽谈会，畅通了特色农产品销售渠道，电子商务从无到有，在农产品营销和促农增收中发挥了积极作用。工业上，加强运行监测服务，支持重点企业生产经营，全市规模以上工业增加值完成40.2亿元，同比增长19%。市列32个重点工业项目建设进展良好，陇南紫金矿业日处理6000吨矿石、西和后川坝金矿日处理1000吨矿石生产线扩建等12个项目已经建成。全市新增规上工业企业21户。全市工业用电量比上年增长27.3%。万元工业增加值能耗和工业用水量分别下降9.7%、6.3%。第三产业上，加强旅游与文化的深度融合，启动“1313”陇南特色文化大市建设工程，成功举办中国(西和)乞巧文化高峰论坛和首届“西狭颂”全国书法大展。在深圳文博会、兰洽会和第二届国际文化产业大会上，我市共签约文化产业项目20个，签约资金36亿元，比上年增长78%。旅游宣传取得实效，国内旅游接待人数和旅游综合收入比上年分别增长34.9%和37.5%。继续实施扩大内需政策，商贸流通持续繁荣活跃，市场体系建设进一步加快，三产增加值和文化产业增加值分别增长11%、36%。全市非公经济增加值达到126.7亿元，占全市生产总值的49.7%，增速达到19.3%。

二、项目建设和招商引资取得新进展，群众生产生活条件明显改善

2013年，我们紧紧抢抓各类扶持政策叠加的难得机遇，全力抓好项目工作。重大项目争取工作取得新成效，渭武高速公路开工建设，武九高速、十天高速公路两当连接线等重大项目前期工作进度加快，“两江一水”治理规划获得国家批复，我市被确定为全国首批45个水生态文明城市建设试点之一。在建重大项目稳步推进，兰渝铁路和武罐、成武、十天高速公路等9个续建省列重大项目完成投资121亿元，武罐高速建成通车，结束了陇南没有高速公路的历史，成武高速完成路基工程，十天高速进度加快，成州民用机场试验段建设进展良好。“3341”项目和市列重点项目分别完成投资499.6亿元、243.8亿元，世行贷款31个子项目累计完成投资9.6亿元，竣工21项。国省干线和县乡公路改造提升全面推进，礼县至武都、洛门至礼县、宕昌南河至迭部腊子口公路、五马至阳坝公路等重点项目进展顺利，全市建成农村公路通畅工程1000公里，100个不通公路建制村实现通达目标，礼县、两当通村公路建设试点工作取得较好进展。城镇建设力度进一步加大，全市实施城镇市政基础设施项目156项，市区框架进一步拉大，东江、钟楼滩、吉石坝等新区开发建设取得突破性进展，长江大道东西延伸一期工程全线贯通，各县县城路网改造、供水供热、垃圾污水处理等重点市政项目加快推进，城市品位进一步提升。两当县被评为全国园林化县城。城镇化综合改革试点、智慧城市试点工作启动实施。全市城镇化率达到25.6%。开工建设各类水利项目345项，290个农村饮水安全工程进展良好，解决了25.3万农村人口和2.8万学生的安全饮水问题，实施了一批江河支流及中小河流治理项目，新修堤防160公里，新增高效节水灌溉面积4.45万亩，新修梯田18.5万亩，新增水土流失治理面积200平方公里。生态治理、环境保护和林业生态建设取得新成效，污染减排和重金属污染治理扎实有效开展，58个农村环境连片整治项目顺利推进。能源和信息化建设得到加强。招商引资取得新突破，市上组织党政考察团多次赴国家部委、长三角地区和新疆等地争取项目，学习考察，谋求合作，与无锡、金华两市签订了经济合作协议，与义乌、永康、石河子、阿拉尔等市建立了友好合作关系，与一批大型企业集团达成初步意向，衔接落实了一批重点招商引资项目。在第十九届兰洽会上，我市共签约招商引资项目125个，签约资金376.6亿元，比上届增长一倍。在上海成功举办招商引资项目推介会暨签约仪式，签约项目21个，签约资金74亿元。全市全年共签约招商引资项目179个，签约资金423亿元，到位资金180亿元，同比分别增长78.5%、97.2%。

三、扶贫开发力度进一步加大，双联行动取得新成效

围绕实现2017年整体脱贫的目标，制定出台了关于深入推进扶贫攻坚行动的实施意见，调整市、县领导和部门联系包抓贫困村方案，进一步突出扶贫重点，加大帮扶力度。在全市筛选确定25个特困片区、80个特困乡镇、822个特困村，作为扶贫攻坚主战场。积极争取秦巴扶贫规划项目资金，全年争取财政扶贫资金3.45亿元，实施县级扶贫连片开发9个、扶贫整村推进项目138个，整体形象进度达到95%以上。建成生态文明新农村371个。我们把劳务输转作为增加农民收入，加快脱贫步伐的最有效途径，不断加大有组织输转力度。扎实开展“两后生”和雨露计划培训，完成职业技能培训2.5万人，职业技能鉴定1.5万人。与新疆生产建设兵团的劳务合作取得新进展，组织输送劳务人员8640人，超额完成省上计划目标，输送“双五千”人才1254人。全市输转劳务74万人次，创收96亿元。科学规划，积极推进，实施易地扶贫搬迁1万人。全市减少贫困人口25万人。双联行动深入开展，市级领导和市直单位增加联系特困村100个，各联村单位投入各类资金16.9亿元，为联系村帮办各类实事2.2万件，一些群众生产生活的实际困难得到解决。

四、财政金融工作迈上新台阶，服务发展能力得到提升

进一步健全收入征管机制，全力组织财政收入，切实保障重点支出。全市大口径财政收入和公共财政预算收入均实现快速增长，同比分别增收10.2亿元和5.48亿元。全市完成公共财政预算支出160亿元，增长9.1%，增支13.3亿元，有力保障了项目建设、民生工程和社会事业等各类重点支出。金融工作取得突破性进展，全市金融机构贷款余额、存贷比等主要指标均有显著提高，在加强银企对接和帮助农民大幅度增加小额贷款等方面取得突出成效，全市新增贷款62.9亿元，同比增长51.7%，新发放涉农贷款36.2亿元，占新增贷款的57.5%，发放双联惠农贷款1.0397万户7.84亿元。新发展村级资金互助协会109个。金融服务体系建设得到加强，甘肃银行建设路支行正式挂牌营业，成立了全省首家民间融资中介机构，组建成立市公共资源交易中心和市龙江城乡发展有限公司，累计建成小额贷款公司14家、融资性担保机构11家。直接融资和间接融资均提前超额完成全年任务，完成企业直接融资11.5亿元。

五、社会各项事业协调发展，民生建设得到加强

加快发展教育事业，认真落实教育惠民政策，全年教育专项支出9.6亿元。各级各类学校贫困学生资助体系逐步完善，农村义务教育营养改善计划全面落实，惠及农村学生23万名。教师队伍建设不断加强，全市新补充教师1432人。教育质量得到提升，全市普通本科上线人数比上年增加193人。市一中创建省级示范高中已通过省上初评，民办高中教育稳步发展。学前教育发展迅速，全市创建省级示范性幼儿园4所。陇南师专、陇南农校、成县职业中专三校资源整合，为进一步发展高等教育打下了良好基础。科技创新和成果转化力度加大，争取到省、部科技计划项目28项，获得全省科技进步一等奖1项，实现了我市获省级科技最高奖项零的突破。广播电视网络整合工作进展顺利，文化惠民工程取得实效。医药卫生体制改革深入推进，公立医院综合改革试点工作有序开展。农村卫生工作得到加强，村卫生室标准化建设取得新进展，全市新农合参合率达到97.5%，新农合筹资标准达到每人每年340元。基本公共卫生服务均等化项目管理水平显著提升。康县创建全省中医药工作先进县，两当县争创国家级卫生县城，西和县、礼县创建省级卫生县城工作均已通过省上考核验收。康县、宕昌、文县创建国家级计划生育优质服务先进县通过评估验收。坚持把改善民生作为政府的重要职责，与群众生活密切相关的民生支出达131亿元。社会保障和救助水平进一步提高，农村低保动态管理机制基本建立，城乡低保标准分别提高15%和28%，农村五保供养年人均补助标准提高到2600元，城乡居民社会养老保险覆盖面提高到97.5%，基础养老金提高到每人每月60元。全面落实就业扶持政策，全市城镇新增就业人数超额完成省政府下达任务。实施农村危旧房改造1.98万户，新建保障性住房926套。省上确定的10项26件和市上确定的10件民生实事全部办结。严格落实安全生产责任，主要指标控制在省定目标之内，安全生产形势基本稳定。物价监管和食品药品安全监管措施全面落实，群众饮食和用药安全得到有效保障。更加注重社会稳定源头防控，我市社会稳定风险评估工作得到中央维稳办充分肯定，在全国社会稳定风险评估经验交流会上介绍了经验。扎实开展矛盾纠纷排查调处和突出信访问题集中治理工作，排查化解各类矛盾纠纷8294件，上访批次和人次分别下降42%和31.8%。大力发展见义勇为事业，成县荣获全国见义勇为工作组织奖。积极构筑社会治安防控体系，深化平安陇南建设，深入推进社会管理综合治理，建成乡镇社会服务管理中心179个，村级社会服务管理站2159个，社会大局保持和谐稳定。民族宗教、外事侨务、统计、审计、档案、地方志、气象、“双拥”、人防、民兵预备役和妇女儿童、残疾人、老龄、慈善、红十字会等各项事业取得新进步。

六、受灾群众生活得到妥善安置，抗灾救灾和灾后重建取得阶段性成效

2013年，我市面对连续遭受干旱、低温霜冻、暴洪、地震等多种自然灾害的挑战，市委、市政府科学应对，靠前指挥，组织全市广大干部群众齐心协力抗灾救灾，全力搜救遇险人员，及时转运救治伤员，妥善转移安置受灾群众，迅速抢修受损基础设施，有效防范次生灾害。一些基层干部急群众之所急，帮助群众排险自救，恢复生产，涌现出一批可歌可泣的感人事迹。全市应急抢险和抗灾救灾取得显著成效，交通、供电、供水、通讯等基础设施在较短时间内迅速恢复，灾区群众临时居住和生活困难问题得到及时解决，越冬生活得到妥善安置，保证了受灾群众有饭吃、有干净水喝、有临时住所。防灾减灾预警体系发挥了应有作用，由于预警、转移、避险及时有效，在20次强降雨和9次大暴雨等灾害面前没有发生直接人员死亡，把灾害损失降到了最低程度。适时启动灾后重建，加快编制灾后重建规划，全市共规划灾后恢复重建项目5303项，争取到地震灾后重建中央及行业补助资金18亿元，暴洪灾害救灾及重建补助资金1.59亿元，灾后重建工作正在有序推进当中。

在全面推进各项工作的同时，在市委的坚强领导和组织下，圆满承办了五次国家和省级重要会议及活动。

各位代表，过去一年的成绩来之不易。这些成绩的取得，是省委、省政府关怀支持的结果，是市委正确领导、科学决策的结果，是市人大、市政协和社会各界监督支持的结果，是全市人民艰苦奋斗、顽强拼搏的结果。在这里，我代表市人民政府，向奋战在各条战线上的广大干部群众，向驻陇南人民解放军、武警官兵和中央、省驻陇南单位，向各民主党派、工商联、无党派爱国人士、离退休老干部，向奋战在陇南的重大建设项目施工人员，向所有关心支持陇南经济发展、社会进步的各界人士表示衷心的感谢和崇高的敬意！

一年来工作的实践和取得的成效给予我们许多有益的启示，我们深切体会到，要加快陇南经济社会跨越发展，实现与全国全省同步建成全面小康的目标，必须坚持以党的十八大和十八届二中、三中全会精神为指引，认真落实省委、省政府和市委的重大决策部署，牢牢把握发展方向，坚持发展不动摇，把促发展作为长期的战略任务和神圣职责；必须坚持历届班子和人民群众在实践中探索和积累的成功经验，坚持实践证明行之有效的发展措施，在继承中完善，在创新中发展，使我们的发展战略和工作思路更加符合陇南实际，更加符合群众期待；必须坚持深化改革、扩大开放，以更坚强的决心、更大的气魄推进各项改革，大力推进体制机制创新，增强发展的内生动力；必须坚持民生优先，不断改善人民群众的生产生活条件，以扶贫攻坚为统揽，在发展富民产业上求突破，使人民群众更多更公平地享受发展成果。

在充分肯定成绩的同时，我们也清醒地认识到，经济社会发展还面临着许多矛盾和问题。主要表现在：保持固定资产投资增速的难度大，且投资渠道单一，多元投资的能力不强；受交通、环境等诸多因素的影响，招商引资项目落地率不高，工业园区建设的步伐有待加快；农业产业化程度低，农业增产和农民增收难度大，转方式、调结构任务艰巨；自然灾害易发频发，防灾减灾的压力仍然很大；部分工业企业，特别是铅锌企业经营困难，效益下降，非公经济规模小、比重低；第三产业增长乏力，文化旅游发展缓慢，科技的支撑作用有待进一步发挥；发展环境有待进一步改善，政府服务发展的能力和水平有待进一步提升。

2014年主要工作任务

各位代表，2014年是贯彻落实党的十八届三中全会精神，全面深化改革的第一年。今年政府工作的总体要求是：以党的十八大和十八届二中、三中全会精神为指导，按照省委、省政府重大决策部署和市委“433”发展战略，坚持用改革统揽政府工作全局，坚持有所为有所不为，扬长避短、后发赶超，在扶贫开发、生态文明、产业培育和城乡一体等方面快速推进，在基础设施、改善民生、社会治理等方面着力夯实，在电子商务、金融支撑、非公经济等方面集中突破，努力推动经济社会跨越发展。

经济社会发展的主要预期目标是：生产总值增长11%；固定资产投资增长25%；规模以上工业增加值增长17%；财政收入增长13%；社会消费品零售总额增长14%；城镇居民人均可支配收入增长13%；农民人均纯收入增长17%；城镇登记失业率控制在4%以内；居民消费价格涨幅控制在3.5%以内；万元生产总值能耗和主要污染物排放控制在省定目标以内。

为了确保完成全年各项目标任务，重点抓好以下工作：

一、以扶贫开发为统揽，全力抓好农业农村工作

坚持以持续增加农民收入为核心，认真实施秦巴扶贫规划和“1236”扶贫攻坚行动，加快农村经济提质增效和跨越发展。实现农业增加值增长6.5%。一是改善贫困地区生产生活条件。瞄准25个特困片区、80个特困乡镇、822个特困村，作为扶贫攻坚的主战场，科学规划，整合资源，加大投入，加快实施一批水电路房田等基础设施项目。全市贫困村动力电覆盖率达到97.8%以上，自来水入户率达到62%以上。确保完成210个生态文明新农村建设任务。完成易地扶贫搬迁1.5万人。建立政府主导、社会帮扶和群众主体的扶贫攻坚机制，落实市县领导责任，深入开展双联行动，扩大双联惠农贷款、妇女小额贷款发放，加快发展村级资金互助组织，推动扶贫开发取得新成效，全市减少贫困人口20万人。二是加快农业特色产业提质增效。深入实施“365”现代农业发展行动，加快规模化基地建设，新发展特色农业25.5万亩，规模化基地比重达到60%以上。抓好核桃、花椒、油橄榄等12个特色现代农业示范区建设。积极引进试种油用牡丹。加快发展畜牧业规模化养殖，畜牧业增加值增长10%以上。加大良种引进、选育和推广力度，积极开展以嫁接换优和病虫害防治为主的经济林综合管理。进一步推动农业科技人员与农村合作组织和种植养殖大户深度融合。加大土地流转力度，新增土地流转面积30万亩。大力支持和培育农民专合组织、家庭农场、专业大户、龙头企业等农业新型经营主体，推进特色产业规模化经营。大力实施品牌战略，加强特色产品认证和商标注册工作，整合茶叶产业品牌，加快陇南油橄榄产业园建设，抓好国家油橄榄工程研发中心建设。强化农产品质量安全监管，确保农产品质量安全。加强农产品营销工作，办好核桃、花椒、中药材、苗木等特色农产品产销对接洽谈会，发挥好促农增收的作用。启动美丽乡村建设，把“农家乐”、“乡村游”作为农民增收的重要渠道。三是加大劳务输转力度。争取输转劳务73.3万人。加强劳务技能培训，整合培训资源，不断扩大“两后生”培训规模，完成“两后生”职业技能学历教育培训7000人，开展劳动力技能培训10万人以上。根据劳务基地用工和人才需求，调整职业技术学校专业设置，与用工地开展联合办学和订单培训，提高职业技能培训的针对性和实效性，培养更多符合输出地专业需求的技术人才，增加农民务工收入，使贫困农户工资性收入占比达到70%以上。四是抓好粮食生产。加强农业综合管理和农业技术服务体系建设，全面落实科技增粮措施，推广全膜双垄沟播技术29万亩、高效农田节水技术28.9万亩，扩大粮油高产创建示范面积，力争粮食总产稳定在104万吨以上。

二、以产业培育为重点，不断提升工业经济总量和效益

继续加大矿产资源和水能资源、建筑建材、有色冶炼、农产品精深加工、中医药、文化旅游的产业培育和建设，在整合资源、做大企业规模上下功夫；在提高运行质量、改造升级，做强企业实力上下功夫；在招商引资、多元发展，寻求新的工业增长极上下功夫。一是抓好工业项目建设。建成两当中金日处理800吨矿石生产线、成县200万吨干法水泥生产线等重点项目，苗家坝、橙子沟、沙湾电站蓄水发电。加快推进厂坝公司300万吨矿山扩能技改、金徽矿业郭家沟铅锌矿日处理5000吨矿石生产线等亿元以上项目，开工建设陇南紫金二期日处理6000吨矿石生产线、西和中宝矿业4000吨矿石生产线等重点项目。实施一批传统产业技术改造升级项目，加快煤矿企业兼并重组，推动煤炭资源规模化、集约化开发。论证和贮备一批重点工业项目，为扩张工业经济总量奠定基础。二是加大重点企业扶持力度。加强重点企业监测分析，西和县大桥金矿等建成项目尽快达产达标。加强规上企业培育和申报工作，力争新增入规企业9户以上。积极扶持小微企业发展，协调解决项目审批、用地、资金、环保等问题，为企业发展创造良好环境。鼓励企业推进科技创新，提高节能降耗和污染减排水平，增强循环利用和可持续发展能力。优化资源配置，把矿产、水力资源更多地向大型企业集团配置。落实水能资源开发利用管理办法和矿业权审查管理暂行办法，提高资源配置效率和公平性。三是大力推进工业园区建设。加快西成经济开发区、徽县工业集中区扩区工作，抓好核心区基础设施建设，加大招商引资力度，力争核心区建设取得明显突破。抓好各县区园区基础设施建设和规划报批，加强企业入园和项目落地工作。对工业园区建设进行单独考核。四是加快发展非公有制经济。把发展非公经济放到更加重要的位置，全面落实放宽市场准入的政策措施，着力消除非公经济发展中的“玻璃门”“弹簧门”“旋转门”。制定和落实优惠政策，鼓励非公资本参股国有资本投资项目，支持非公经济平等参与境内资源开发，投资文化产业和旅游景区开发，允许非公经济参与城镇基础设施建设，支持非公经济参与发展学校、医院、市政设施、公共交通等涉及公共资源配置的社会民生事业，引导、鼓励非公经济投资生产加工等实体经济领域。鼓励财政资金通过贷款贴息、担保、抵押等方式扶持非公经济发展。解决好非公企业土地供应、融资和优惠政策落实等问题，在矿产、金融、运输以及其他资源配置方面，对非公经济与其他所有制经济一视同仁，激发全民创业，增强经济内生活力。

三、以城镇建设为重点，大力推进城乡一体化进程

破解城乡二元结构，推进以人为本的城镇化建设，统筹推进市区、县城、建制镇、新农村四位一体建设，让广大农民平等参与现代化进程、共同分享现代化成果。一是加快构建城乡规划体系。坚持城乡统筹、产城联动、要素聚集，加快推进村镇规划编制，全面完成乡镇规划编制，抓好历史文化名镇名村规划编制，使全市乡镇规划编制达到100%，村庄规划编制达到70%。严格执行总体规划和控制性详规，确保城镇建设高标准高水平推进。二是统筹推进城乡发展。完善城镇化健康发展的体制机制，推动县城和小城镇协调发展、产业和城镇融合发展、城镇化和新农村共同发展。积极创造条件，争取一部分条件具备的乡改为建制镇。稳步提升城镇基本公共服务功能，增强以城带乡的能力，推进农业转移人口市民化，促进农民向城镇合理有序流动。力争全市城镇化率达到27.6%。三是加大城镇基础设施建设力度。加快市区东江新区、钟楼滩新区、吉石坝新区和长江大道东西延伸段建设，建立市区联动机制，建设火车站站前广场、商业综合广场、滨江体育场改造、阶州古街等10个重点城市建设项目。抓好八县县城道路、供排水、垃圾污水处理等市政基础设施项目，完善城镇功能。强化社区和住宅小区管理，加强城区环境卫生整治，提高城镇管理水平。新增城镇集中供热面积125万平方米，新建城市供水管网33公里、雨水管网60公里，城镇污水处理率、生活垃圾无害化处理率分别达到60%以上、40%以上。四是开展小城镇综合改革试点工作。在全市选择条件具备的9个重点小城镇，纳入“百镇千村”建设工程，用深化改革的办法，把小城镇建设与新农村建设、易地搬迁、危旧房改造、保障性住房建设相结合，努力破解扶贫开发难题，二元结构难题，资源整合难题和融资难题，让小城镇承担大产业，赋予新内涵，增强小城镇吸纳农业人口、加快扶贫开发的带动能力，打造一批有历史记忆、地域特点、民族特色，宜居、宜业、宜游的陇上江南特色文化小镇。以试点镇建设带动示范镇和公路沿线重点乡镇建设。结合小城镇改革试点，探索乡镇政府小城镇管理职能和方法。逐步探索建设农民物权交易平台，真正让农民享受到更多财产权，拓宽农民增加财产性收入的渠道。

四、以林业建设和环境保护为重点，扎实推进生态文明建设

坚持人与自然和谐相处，节约资源，谨慎开发，多措并举，有效保护，走出一条生产发展、生活富裕、生态良好的新路子。一是全力实施好生态文明示范市工程。突出抓好“两江一水”区域综合治理，统筹推进整片整流域建设。全面抓好水源、森林、湖泊、湿地等生态资源保护，加强风景名胜区保护工作。积极争取国家把陇南纳入生态补偿试验区。深入实施小流域综合治理、生态修复、天然林保护、公益林补偿、水土流失治理、梯田建设等工程，加大荒山、荒坡、弃耕地恢复治理，抓好重点流域、重点区域植被恢复，巩固退耕还林成果，争取启动实施新一轮退耕还林工程和长防林三期工程。完成造林30万亩以上，建设绿色通道400公里，落实好天然林管护措施。二是深入开展环境污染治理。加大河流、大气污染治理力度，认真落实污染减排措施，加强重点区域污染防治和排污企业综合治理，抓好西和县重金属污染治理示范县建设，推动重金属污染治理取得新成效。深入开展环保专项行动，严厉整治各类环境违法行为。严格落实主体责任，加大矿山、尾矿库治理恢复力度。加快发展循环经济，大力推进节能降耗，实施好重点节能改造项目，加快西成经济开发区循环化改造。三是着力改善农村人居环境。加强农村环境综合整治工作，抓好康县美丽乡村试点县和10个示范村建设，建设150个环境整洁村。加大农村面源污染治理力度，推进废旧农膜回收利用。四是进一步抓好地质灾害综合治理和防灾减灾工作。全力抓好陇南山地国家级地质灾害防治管理及监测预警示范区建设项目。加快实施西汉水、白龙江、白水江、嘉陵江河段综合治理工程，建成覆盖市区和八县县城主要河流、沟道的城市防汛视频远程监控系统。加快实施地震预警项目，完善地震监测网络，提高地震监测预报水平。建成新一代天气雷达监测系统，力争今年主汛期投入试运行。

五、以项目建设为载体，着力提升基础设施支撑能力

围绕贯彻“3341”项目工程，抢抓国家支持西部地区基础设施建设特别是铁路、公路、水利建设的机遇，抓好政策运用和项目争取工作，在项目实施和基础设施建设上取得新突破。一是加大项目争取力度。进一步抓好向国家、省上汇报争取工作，重点争取带动性强、有利于陇南整体发展的项目，抓好已经汇报项目的跟踪衔接，确保国家部委、省直厅局拟建项目对陇南的支持。做好省政府《意见》和秦巴扶贫、“两江一水”治理、生态示范市等重大政策规划的项目衔接工作，努力把各类政策规划变成项目和资金。抓住国家支持中西部地区铁路建设的机遇，加快天平铁路南延线、阳平关至武都至九寨沟铁路项目的前期工作进度。力争早日开工建设十天高速两当连接线和武九高速公路。二是加快在建重大项目建设。加强重大项目协调服务，妥善解决拆迁占和施工保障等问题，全面加快兰渝铁路、成武、十天、渭武高速和成州民用机场建设进度，实现成武高速建成通车，新增高速里程91公里，兰渝铁路基本完成建设任务，为2015年通车创造条件，十天高速完成工程量的70%，确保2015年建成通车，成州机场、渭武高速完成年度投资计划。加快世行贷款项目实施进度，确保按期完成建设任务。三是加强基础设施建设。根据甘肃省国家公路网线位规划，积极争取连接线项目，使地方公路与国家公路便捷连接，提升公路网络化水平。抓好国省干线、县乡公路改造升级和出口路、联网路建设，建成宕昌南河至迭部腊子口、洛门至礼县、五马至阳坝等联网公路，加快礼武公路建设进度，年内完成路基工程，开工建设礼县至康县公路、国道212线峰岭至罐子沟升等改造工程。加快实施建制村通畅工程，建设通村水泥路3000公里，使建制村公路硬化率达到60%。抓好礼县、两当通村公路建设试点工作，建制村公路硬化率分别达到70%、76%，争取省上下达新的试点任务。加快实施农村饮水安全、江河主要支流及中小河流治理、磨坝峡水库、武都区中央财政小型农田水利重点县等项目，争取早日启动陇南市山丘区特色农业节水灌溉示范区、50万亩油橄榄水利配套项目和陇南市水生态文明城市建设试点工作。完成水土流失治理200平方公里，新修梯田14.8万亩。加快能源和信息化建设，完成信息基础设施投资3.5亿元，积极发展4G通讯，抓好智慧城市试点工作。四是进一步加大招商引资力度。落实招商引资工作责任制，完善优惠政策，进一步优化招商引资环境，统筹推进项目引进和落地工作。加强与有合作意向企业及地区的衔接洽谈，抓好与大企业、大集团的产业对接，有针对性地做好项目推介和招商引资工作。特别是进一步加强与长三角地区和相关友好城市的交流合作，不断拓宽合作领域，引进更多的大型企业和投资项目。加强已签约项目的跟踪衔接，解决好影响项目落地的具体问题，切实提高项目落地率和成功率，实现招商引资到位资金234亿元以上。

六、以大力发展电子商务为突破口，努力提升第三产业发展水平

坚持现代服务业和传统服务业并重并举，加强商贸流通，完善市场体系，全面推动支撑三产的各类重点行业快速发展，努力破解制约第三产业发展的瓶颈问题。一是全力扶持发展电子商务。把电子商务作为畅通产品销售渠道、扩大社会就业、提高陇南知名度的重要途径，出台鼓励支持电子商务发展的政策措施，在全市大力发展以农特产品网上交易为重点的电子商务。加强对乡村干部特别是大学生村官、两后生、经合组织成员的培训，建立一批电子商务供应商、服务商和网商队伍，推进电子商务向各个商业领域拓展。率先在有条件的县区建立电子商务发展中心和产业园区，鼓励支持各县区和商会组织建设财富中心、会展中心和电商孵化器，加快构建全市农产品电子商务营销体系，把电子商务建设成为农特产品销售的重要平台。二是着力加快重点行业发展。制定完善优惠政策，积极扶持批发、零售、餐饮、住宿、运输等传统服务业的发展，放手放胆支持信息、物流、仓储、配送、会计、咨询、中介服务、娱乐消费等现代服务业，降低门槛，优化服务，营造良好发展环境。积极培育信息消费等新的消费增长点，增强消费对经济增长的拉动作用。三是加快完善现代市场体系。大力发展商会组织和饭店、餐饮、服装、物流等各种类型的协会组织，充分发挥协会、商会在调节市场、促进要素流动、推进产业升级中的作用。加强流通体系和商贸设施建设，培育、引进大型连锁商贸企业，全市城乡连锁化率、商品统一配送率分别达到90%、92%。继续实施好“新网工程”，抓好以危旧粮库改造为主的仓储设施建设，加强粮食购销储备安全管理和流通体系预警监测。加快陇南市特色农产品交易市场、宕昌哈达铺物流中心和武都吉石坝物流园区建设，建成花椒、核桃、茶叶、中药材、苗木等专业市场。加快建立市级三权交易平台，提高资源配置市场化程度。

七、以华夏文明传承创新区建设为平台，加快文化旅游产业发展

大力推进华夏文明传承创新区特色文化大市建设，推动文化旅游产业深度融合发展。文化产业投资达到9亿元，文化产业增加值增长30%。一是着力打造文化品牌。坚持多中选好、好中选优，集中力量打造以秦西垂陵园为代表的秦文化品牌、以哈达铺红军长征和两当兵变为代表的红色文化品牌、以乞巧为代表的民俗文化品牌，形成陇南文化发展的重要支点。加快各县区红色革命纪念馆建设，努力延伸红色文化产业链。争取把中国西和乞巧女儿节申办成国家级节会。二是大力发展文化产业。按照打造“6551”文化产业体系的要求，论证规划和实施一批文化产业项目。加强文化产业项目招商引资工作，抓好31个已签约项目的落地实施。积极支持市内文化企业，引进一批大型文化企业，发挥好文化企业的主力军作用。三是加快旅游大景区建设。创新旅游开发机制，组建市县文化旅游投融资平台，发挥财政资金引导作用，以市场化运作方式推进旅游资源开发。启动实施大景区规划建设，打破行政区域限制，合力打造独具特色的红色文化旅游、生态体验旅游、历史民俗旅游、养生度假旅游等重点景区。深入挖掘景区景点文化内涵，把文化元素融入到规划编制、景区打造、产业开发等具体环节中。协调金融机构加大对旅游开发企业的授信额度，吸引民间资本、社会资本投资文化旅游，解决旅游景区开发投资难问题。创新景区景点建设方式和管理模式，创新旅游富民的思路和措施，让更多的农民通过发展旅游产业增收致富。加快发展中医养生养老保健旅游产业，积极打造中医养生养老保健旅游示范区。加快建设旅游名镇和旅游示范村，力争创建国家4A级景区工作取得新进展。加强与周边地区旅游协作，打造与国内热线相衔接的文化旅游线路。大力发展智慧旅游，积极利用新闻媒体开展旅游营销，利用电子商务平台销售旅游产品，加快推广移动互联技术，在大景区全面提供WIFI、二维码导览等服务，努力提升旅游服务综合水平。四是做好文化资源保护工作。抓好文化资源普查。积极做好“陇蜀古道”申遗工作，争取进入世界文化遗产名录。实施以《西狭颂》等碑刻文化及石窟文化、古建筑、古遗迹保护为重点的文物保护项目。加强秦早期文化大遗址、大堡子山墓群、青泥岭古道遗址、三国古文化遗址等保护建设工作。加强非物质文化遗产保护传承，抓好宕昌哈达铺、文县碧口镇、哈南寨、两当杨店等历史文化名镇名村保护开发工作。五是扎实推进文化惠民工程。深入实施广播电视“户户通”、文化信息资源共享、农村电影放映、农家书屋等文化惠民工程，抓好农村舞台建设，完善乡镇文化站、农村文化大院、文化广场配套设施，建设氐羌文化、乞巧文化、西狭文化等一批特色博物馆，不断提高公共文化服务水平。

八、以提升金融支撑能力为突破口，加强财政金融工作

通过扶持企业发展、争取项目资金、调整产业结构，逐步形成支撑有力、后续强劲、多元化的财源体系。加强重点税源监管，加大主体税种、零散税种和非税收入征收力度，确保财政收入稳定增长。优化财政支出结构，严格落实厉行节约各项规定，集中财力保障项目建设、民生工程和社会事业等重点支出。完善金融服务体系，鼓励具备条件的民间资本依法发起设立中小型银行等金融机构，通过政府参股、引进民资等形式组建注册资本规模在5000万元以上的融资性担保公司，积极引进兰州银行在陇南设立分行，鼓励有实力的商业银行在陇南设立村镇银行，稳步有序发展小额贷款公司、民间融资服务中介等新型金融主体。大力发展普惠金融，加快“三权”确权工作，积极推进“三权”抵押贷款试点，破解农户抵押、担保难问题。抓好银企对接活动，加大金融对实体经济特别是中小微企业的支持力度，帮助中小微企业解决发展资金难题。创新财政补贴方式，把财政直补资金转变为政策性贷款贴息、融资担保基金和风险补偿基金，放大信贷总量，为企业和低收入群体提供良好金融服务，力争全市各类扶贫贷款高于全市贷款平均增幅。协调各银行业机构加大信贷投放，力争全市金融机构存贷比提高2个百分点。加强政府融资平台建设，加快组建市县区扶贫开发公司，力争陇南市龙江城乡发展有限公司实现5-10亿元融资计划，着力破解重点项目融资难题。加快构建多层次资本市场，力争金徽酒业上市融资。

九、以保障和改善民生为出发点，统筹发展各项社会事业

不断深化教育教学改革，创建一批示范学校。进一步优化教育布局，破解农村学校师资短缺问题，推进义务教育均衡发展，进一步提升中小学教育质量。大力发展职业教育，加大职业教育资源整合力度，不断创新职业教育办学模式，创造条件成立陇南职业技术学院。加快发展学前教育，抓好乡镇中心幼儿园建设。积极发展特殊教育，努力让残疾人等特殊群体平等获得受教育权利。引入多元化办学机制，提倡利用社会资源办教育。不断加大科技投入，积极争取实施省部科技项目，进一步抓好科技成果转化应用，鼓励支持企业开展技术创新，积极推进农技部门职能转变。实施好乡镇卫生院标准化建设项目。加强重大疾病防控，落实好妇女儿童保健措施。做好武都区、徽县全省中医药工作先进县创建工作，力争宕昌县通过省级卫生县城考核验收。全面落实计划生育家庭奖励扶助政策，推进优生健康惠民工程，加强人口信息化建设，提高计生基本公共服务均等化水平，积极创建流动人口计生基本公共服务均等化示范市。大力发展群众体育运动，提高竞技体育水平。办好第三届全国业余围棋象棋邀请赛，组队参加甘肃省第十三届运动会。加强体育基础设施建设，建成投用市体校、体育场、体育馆，新建乡镇及社区体育健身中心10个。完善和落实促进就业的扶持政策，加快建设创业示范园区和创业孵化基地，落实小额担保贷款政策及资金，满足城乡劳动者创业需求。实施好各类扶持高校毕业生就业的民生实事项目，力争20%的高校毕业生实现自主创业，争取输送1000名左右高校毕业生到新疆等地就业，全市应届高校毕业生就业率达到85%以上，新增城镇就业1.5万人以上。着力提高社会保障水平，改革创新被征地农民纳入社会养老保险机制，做好城乡低保人群评级调级工作，全面推行城乡低保动态管理，促进最低生活保障政策有效落实。抓好重特大疾病医疗救助试点工作。新建保障性住房1686套，发放城镇困难家庭廉租住房补贴4710户，加快农村危旧房改造，努力解决城乡低收入群体住房困难。着力抓好灾后重建工作，完善重建规划，整合易地扶贫搬迁、农村危旧房改造等项目资金，加快受灾群众住房、基础设施、公益设施、工农业生产设施恢复重建，确保按期完成省政府确定的重建目标。加强灾后重建项目质量监督管理，实行建筑质量终身负责制。及时下拨各类补助资金，切实加强重建资金使用管理，发挥好资金使用效益。全面落实党的民族宗教政策，推动民族乡村经济社会快速发展。加强民兵预备役建设，坚持开展全民国防教育，深化双拥共建工作。重视发展老龄、残疾人、档案和红十字会事业，支持工会、妇联、共青团等群众团体工作，维护好职工、妇女儿童和青少年合法权益。

按照量力而行，普惠共享的原则，集中力量办好10件民生实事：⑴建设9个电子商务示范点。⑵建设17个乡镇客运站、400个农村停靠点。⑶完成400个行政村的通村水泥路建设任务。⑷解决25万农村人口饮水安全问题。⑸实施易地扶贫搬迁1.5万人。⑹高接换优低产核桃400万株。⑺建设生态文明新农村210个。⑻建设标准化村卫生室100个。⑼发展农村生态清洁能源，发放1万台太阳灶、4000台太阳能热水器。⑽完成11个地震暴洪灾害整村重建点的建设。

十、以改革创新为动力，全力维护社会和谐稳定

积极创新社会治理方式，发挥好社会组织、中介机构、基层群众协同参与作用和自治互律作用，更多地运用民主管理和说服教育方式来进行社会治理。围绕建立社会治理体系，整合市县乡相关部门资源，进一步完善自然灾害监测预警指挥系统，建立社会公共服务系统和高效快捷的电子政务办公系统，努力提高社会治理现代化水平。建立畅通有序的诉求表达和权益保障机制，坚持领导干部联系群众制度、下访接待制度和调查研究制度，抓好群众信访和矛盾纠纷排查调处工作，切实解决群众合理诉求和切身利益问题，着力化解信访积案，努力把矛盾纠纷化解在萌芽状态。把社会稳定风险评估作为前置刚性条件纳入审批程序，构建科学规范、覆盖全市的社会稳定风险评估新格局。深入开展法制宣传教育，加快构建打防管控一体化的社会治安防控体系，不断深化平安陇南建设，依法严厉打击违法犯罪，着力提高人民群众安全感。进一步完善网络舆情监管机制，及时回应网民关切，疏导网络民意，发挥网络积极作用。积极推进社区网格化管理，努力实现社会服务管理站村级全覆盖，提高基层社会治理水平。落实各级安全生产责任和各项监管措施，强化基层基础建设，加强工矿企业、道路交通、危险化学品、建筑施工、消防、特种设备等重点行业领域的安全监管，深入开展隐患排查治理和专项整治活动，严厉打击非法违法行为，切实消除安全隐患，确保人民群众生命财产安全。加强价格监测预警，加大市场价格监督检查力度，抓好节日期间物价监管，确保市场价格保持基本稳定。抓好食品药品安全监管，保障好群众的饮食用药安全。

各位代表，全面深化改革，加快陇南转型跨越发展，是时代赋予政府的光荣使命，是人民群众的殷切期望。我们将把深入学习贯彻党的十八届三中全会精神作为首要政治任务，不断加强政府自身建设，以更加奋发有为的精神状态，更加勤勉务实的工作作风，坚定信心，攻坚克难，扎实工作，廉洁实干，为实现与全国、全省同步进入全面小康提供坚强保证。

深化重点领域改革。按照国务院和省政府的要求，深入推进重点领域改革，着力增强发展活力。完善国有资产监管体制，积极推动经营性国有资产集中统一监管，促进国有资产重组整合和优化配置。深入推进部门预算改革，加强预算绩效管理，积极稳妥推进预决算公开，提高财政管理水平。深化科技体制改革，完善科技评价与科技奖励制度。完善文化市场准入和退出机制，推动文化产品和要素在更大范围内流动。深化医药卫生体制和食品药品监管体制改革，稳步推进县级公立医院综合改革试点。加快事业单位分类改革。深化人事制度改革，完善激励机制，激发人才活力。

加快推进职能转变。围绕使市场在资源配置中起决定性作用，加快转变政府职能，深化行政审批制度改革，减少审批事项，正确处理政府与市场的关系，把能交给市场的一律交给市场，把该放的权力放到位，把该管的事务管上手。完成新一轮政府机构改革。积极推进乡镇政府职能转变，更好发挥乡镇政府职能作用。坚持市场配置资源的基本原则，充分发挥市公共资源交易中心的作用，规范交易流程，建立公开、公平、公正的公共资源交易体系。加强重大项目、工程建设、土地征用出让、国有资产处置、公共资源交易等环节的管理，提高资源配置和项目建设水平。

全力抓好大事要事。坚持抓主抓重，集中精力谋大事，抓要事，解难事。全面落实好市委的总体工作部署，落实好经济工作目标责任，落实好人大及其常委会的各项决议、决定，认真办理人大代表建议和政协委员提案。按照底线思维的原则，强力推进保障和改善民生。坚持用改革的办法破解发展难题，努力解决征地拆迁、招商引资落地、重大项目实施、电子政务、信访维稳、安全生产等热点难点问题，不断开创经济社会转型跨越发展新局面。

着力提升行政效能。认真贯彻中央改进工作作风、密切联系群众八项规定和省委、市委各项规定，把落实规定精神与开展群众路线教育活动结合起来，进一步加强作风建设，坚决反对形式主义、官僚主义、享乐主义、奢靡之风，大兴求真务实之风，改进文风会风，精简会议文件，用更多的时间和精力深入基层，深入群众，调查研究，破解难题。进一步推进政务公开，自觉接受群众监督和社会监督。全面落实岗位责任制、服务承诺制、限时办结制、首问责任制和失职追究制，突出责任落实，坚决做到履行职责决不含糊、承诺事项必须兑现、限时事项按时办结，提高政府工作的公信力和执行力。

始终保持清正廉洁。严格执行廉政建设责任制，扎实推进惩治和预防腐败体系建设，健全风险预警和权力运行监控机制。强化对重要领域、关键岗位的行政监察和审计监督，严肃查处各类违法违纪案件，依法治理和纠正损害群众利益的不正之风。加强对公务员特别是领导干部的教育与监督，认真落实领导干部廉洁自律各项规定。全面贯彻落实中央、省上关于厉行节约的有关规定，坚持艰苦创业、勤俭办事，坚决反对铺张浪费，积极推进“三公”经费公开，公费接待、公费出国、公费购车只减不增，一律不新建政府楼堂馆所，树立清正廉洁的良好形象。

各位代表，事业凝聚人心，行动铸就辉煌！站在新的起点，夺取新的胜利，我们信心百倍，众志成城。让我们在市委的坚强领导下，紧盯与全国全省一道全面建成小康社会的宏伟目标，紧紧依靠全市人民，同心同德，锐意创新，扎实工作，奋力谱写建设幸福美好新陇南的新篇章！
